# Microbiome-host interactions in the pathogenesis of acute exacerbation of chronic obstructive pulmonary disease

**DOI:** 10.3389/fcimb.2024.1386201

**Published:** 2024-07-18

**Authors:** Yao Li, Xiaoyan Mao, Pengfei Shi, Zongren Wan, Dan Yang, Ting Ma, Baolan Wang, Jipeng Wang, Jingjing Wang, Rong Zhu

**Affiliations:** ^1^ Department of Respiratory and Critical Care Medicine, Huaian Clinical College of Xuzhou Medical University, Huaian, China; ^2^ Department of Intensive Care Unit, The Affiliated Huaian Hospital of Xuzhou Medical University, Huaian, China; ^3^ Department of Respiratory and Critical Care Medicine, The Affiliated Huaian No.1 People's Hospital of Nanjing Medical University, Huaian, China; ^4^ Department of Respiratory and Critical Care Medicine, Shanghai Pulmonary Hospital, Tongji University School of Medicine, Shanghai, China

**Keywords:** airway microbiome, chronic obstructive pulmonary disease, innate immune system, IFN, TLR, *Rothia mucilaginosa*, *Haemophilus influenzae*

## Abstract

**Objective:**

To explore the underlying mechanisms the airway microbiome contributes to Acute Exacerbation of Chronic Obstructive Pulmonary Disease(AECOPD).

**Methods:**

We enrolled 31 AECOPD patients and 26 stable COPD patients, their sputum samples were collected for metagenomic and RNA sequencing, and then subjected to bioinformatic analyses. The expression of host genes was validated by Quantitative Real-time PCR(qPCR) using the same batch of specimens.

**Results:**

Our results indicated a higher expression of *Rothia mucilaginosa*(*p*=0.015) in the AECOPD group and *Haemophilus influenzae*(*p*=0.005) in the COPD group. The Different expressed genes(DEGs) detected were significantly enriched in "type I interferon signaling pathway"(*p*<0.001, *q*=0.001) in gene function annotation, and "Cytosolic DNA-sensing pathway"(*p*=0.002, *q*=0.024), "Toll-like receptor signaling pathway"(*p*=0.006, *q*=0.045), and "TNF signaling pathway"(*p*=0.006, *q*=0.045) in KEGG enrichment analysis. qPCR amplification experiment verified that the expression of *OASL* and *IL6* increased significantly in the AECOPD group.

**Conclusion:**

Pulmonary bacteria dysbiosis may regulate the pathogenesis of AECOPD through innate immune system pathways like type I interferon signaling pathway and Toll-like receptor signaling pathway.

## 1 Introduction

Chronic Obstructive Pulmonary Disease (COPD) is a heterogeneous disease underpinned by persistent airflow restriction and reacted airway inflammation in response to harmful exposure, resulting in a progressive decline in lung function and respiratory symptoms (Christenson et al., 2022). While inflammation, alpha1-antitrypsin deficiency, and oxidative stress response have long been considered major pathogenic factors of COPD (Barnes, 2020), novel research elucidated that airway microbiome dysbiosis is a critical factor as well (Zuo et al., 2015; Wang et al., 2016).

The lower airway microbiome structure bears difference to stable-stage COPD when COPD is aggravated, usually manifested as a decline in microbial abundance and diversity. Sputum analysis of AECOPD patients usually described the elevated abundance of *Proteobacteria*, *Haemophilus,* and *Staphylococcus* are presented as aggravation factor, *Veillonella* was described as protective factor (Mayhew et al., 2018; Wang et al., 2018; Leitao et al., 2019; Dicker et al., 2021; Su et al., 2022), but the relative abundance of *Firmicutes* in the acute stage contradicted in some studies (Wang et al., 2010; Huang and Boushey, 2015). No comprehensive conclusion relating the pulmonary microbiome in AECOPD has been reached so far.

Besides, microbiome-host interaction might play a crucial role in the pathogenesis of AECOPD (Huang et al., 2014). The expression of host genes concerning inflammation pathways and immune response, especially macrophage, were increased in AECOPD patients (Yang et al., 2022). *In vivo* and *in vitro* studies conducted by Yan Z et al., indole-3-acetic acid derived by airway microbiome, especially *lactobacilli*, mitigates neutrophilic inflammation, emphysema, apoptosis, and lung function decline in COPD patients (Yan et al., 2022). Furthermore, a multi-omic meta-analysis deduced that airway microbiota promoted the biosynthesis of palmitate, homocysteine, and urate in COPD. These compounds enhanced the airway inflammation through the activation of pro-inflammatory agents (Wang et al., 2020).

While the aforementioned research presented notable findings, their sample size is limited. Also, there exists a deficiency in the identification of host genes when COPD patients experienced exacerbation. A larger qualified patients were recruited and categorized into the Acute Exacerbation of COPD group (AECOPD group) and stable-stage COPD group (COPD group) in our protocol. Their sputum specimens were collected for metagenomics Next Generation Sequencing (mNGS) and RNA sequencing, to explore the potential mechanisms by which airway microbiome contributes to AECOPD and prepare for further research and intervention.

## 2 Materials and methods

### 2.1 Materials

This study recruited a total of 57 COPD patients in Huai'an First People's Hospital (inpatient or outpatient), of which 31 patients underwent exacerbation and 26 patients remained stable in the past 6 months. All participants provided consent and underwent thorough gargling procedures thrice using 10ml of 0.9% normal saline each time, before collecting sputum samples following a full rinse of their oral cavities and posterior pharynx wall. These samples were sent to the laboratory for storage in the refrigerator at -80°C for further sequencing within 2 hours. The inclusion criteria were listed: (1) all recruiters required a post-bronchodilator FEV1/FVC ratio that is less than 0.70, an exposure history of risk factors and/or clinical symptoms (Labaki and Rosenberg, 2020); (2) stable COPD patients means that the symptoms of cough, phlegm or wheezing were stable in 6 months; (3) no systemic steroids or antibiotics use in the past 2 months (Faner et al., 2017); (4) a smoking history or current smokers. Patients complicated with other respiratory tract infections, systematic inflammatory diseases, immunodeficiency or malignant tumor were excluded. All the informed consent forms were signed before the specimens were collected. The study was compatible with the Declaration of Helsinki and was approved by the ethics committee of our hospital (Ethics Number: YX-2021–098-01).

### 2.2 Metagenomic sequencing

Samples of sputum collected were liquefied by 0.1% dithiothreitol (DTT) for 20 minutes at 56°C before extraction. The Qubit (Thermo Fisher Scientific) was used for the quantity assessment of samples and NanoDrop (Thermo Fisher Scientific) for the quality assessment. The KAPA Hyper Prep kit (KAPA Biosystems) was used for the preparation of DNA libraries. The constructed DNA library was sequenced 150bp on Illumina NovaSeq 6000 (Illumina). After splitting raw sequencing data with bcl2fastq2, Trimmomatic was used to remove adapter contamination, low low-quality reads, and duplicated and shot (length<36 bp) reads for high-quality sequencing data. By using bowtie2, reads that could be mapped to human reference genome (hs37d5) were subtracted, reads that couldn't be mapped were retained for comparison with microorganism genome database (downloaded from GenBank release 238, ftp://ftp.ncbi.nlm.nih.gov/genomes/genbank/), the matching reads were obtained for further microbial identification and abundance estimating, using Kraken2 version 2.1.0 and Bracken version 2.5.0 respectively. Here we present criteria that were considered positive results of mNGS: 1. the read number≥1 at the species level for *Legionella*, *Mycobacterium*, and *Legionella pneumophila*; 2. non-overlapping read number≥3 at the species level for other bacteria (except for the above three in criterion 1), virus, fungi, and parasite. We generally didn't consider the pathogen in the Negative 'no-template' control (NTC) positive, unless the reads detected were more than 10 times that in the NTC.

### 2.3 RNA sequencing

We used Trizol regent to extract RNA in the sputum samples, RNase H to eliminate ribosomal RNA, and KAPA Stranded RNA-seq Kit with RiboErase (HMR) (KAPA Biosystems) to prepare library that was sequenced on Illumina NovaSeq NGS platforms (Illumina) afterward. We used bcl2fastq v2.19.0.316 (Illumina, Inc.) to perform base calling for the generation of sequence reads in FASTQ format (Illumina 1.8+ encoding). We used Trimmomatic (version 0.36) to perform Quality control (QC), STAR (version 2.5.3a) to map transcriptome, and RSEM (version 1.2.31) to carry out isoform and gene-level quantification. We used R packages DESeq2 (version 1.22.2) to conduct differential expression analysis and select Differentially Expressed Genes (DEGs) following the principles below: Log2 Fold Change > 2 and P value < 0.05. We used in-house R scripts to plot associated volcano plots and heatmaps, and KOBAS (version 3.0) to conduct GO and KEGG enrichment analysis.

### 2.4 Quantitative real-time PCR amplification experiment

TRNzol total RNA extraction reagent was used for sample RNA extraction; NanoDrop® ND-2000 was used to determine RNA concentration and purity; denatured agarose gel electrophoresis was used to detect RNA integrity; reverse transcription was used to synthesize cDNA; Real-Time PCR was used to detect 3 multiple pores in each sample.

### 2.5 Statistics analysis

Quantitative data that displayed normal distribution and homogeneity of variance was expressed as (x ± S). To compare averages between the two groups, we used a two-independent sample t-test. When the data did not display normal distribution, we used the Mann–Whitney U-test, and data was expressed as Median(M). Qualitative data were expressed as percentages. To compare the data between groups, we used the Pearson chi-square (χ2) test, and when necessary, Fisher's exact probability test (SPSS 25.0, IBM Inc). Statistical analysis was performed by R software (v4.0.1). Differential relative abundance of taxonomic groups at the genus level among groups was tested by using Kruskal-Wallis rank sum test (R package "kruskal.test"). The genera with mean relative abundances greater than 1% and penetrance greater than 40% among all samples were compared. Spearman's correlations between the clinical features and the relative abundances of genera, as well as the DEGs and the relative abundance of species were calculated by the R package "cor.test", and FDR correction was adopted to adjust all p values. The comparison of host genes used Student's t test or non-parametric test(Graphpad Prism 9).

## 3 Results

### 3.1 Demographic data

This study recruited 31 AECOPD patients and 26 stable COPD patients, whose sputum samples were collected and analyzed to seek discrepancies in their microbial composition. The demographic data was summarized in **Table 1** (original data can be found in **Supplementary Material 1**), with patients' age ranging from 42 to 87 years. Male smokers account for a higher proportion of all recruits. No statistical difference of age, sex, Body Mass Index(BMI), Nutritional Risk Screening(NRS) 2002, FEV1/FVC, Inhaled Corticosteroids(ICS) rate, and Exacerbation Frequency Over the Past Year was observed. Smoking Index(*p*=0.040), COPD Assessment Test(CAT) score(*p*<0.001) and mMRC questionnaire(*p*<0.001) increased in the AECOPD group, and FEV1(%pred) (*p*=0.030) decreased in the AECOPD group. GOLD classification(*p*=0.042) and GOLD Groups(*p*=0.029) differed as well.

**Table 1:** General demographic data between AECOPD group and COPD group.

	COPD (N=26)	AECOPD (N=31)	p-value
Age, mean ± SD	68.54 ± 8.00	72.00 ± 8.48	0.121
Sex, Male, n (%)	23(88.5)	28(90.3)	1
Smoking Index, M	500	800	**0.040**
BMI, M	23.1	22	0.059
NRS2002, M	1.5	2	0.057
CAT Score, M	12	23	**<0.001**
mMRC Questionnaire, M	2	3	**<0.001**
FEV1/FVC (%)	51.38 ± 11.94	46.58 ± 10.91	0.119
FEV1(% pred), M	43.5	33	**0.030**
GOLD, n (%)			**0.042**
1	1(3.8)	1(3.2)	
2	8(30.8)	3(9.7)	
3	14(53.8)	14(45.2)	
4	3(11.5)	13(41.9)	
Groups, n (%)			**0.029**
A	9(34.6)	3(9.7)	
B	9(34.6)	9(29.0)	
E	8(30.8)	19(61.3)	
Inhaled Corticosteroid, Y (%)	15(57.7)	24(77.4)	0.111
Exacerbation Frequency Over the Past Year			0.220
≥2, n (%)	8(30.8)	19(61.3)	
0–1, n (%)	18(69.2)	12(38.7)	

The bold values mean statistical significance.

BMI, Body Mass Index; NRS, Nutritional Risk Screening 2002; ICS, Inhaled Corticosteroid; CAT, COPD Assessment Test score.

### 3.2 Overall microbiota compositional profiles

**Figure 1:**
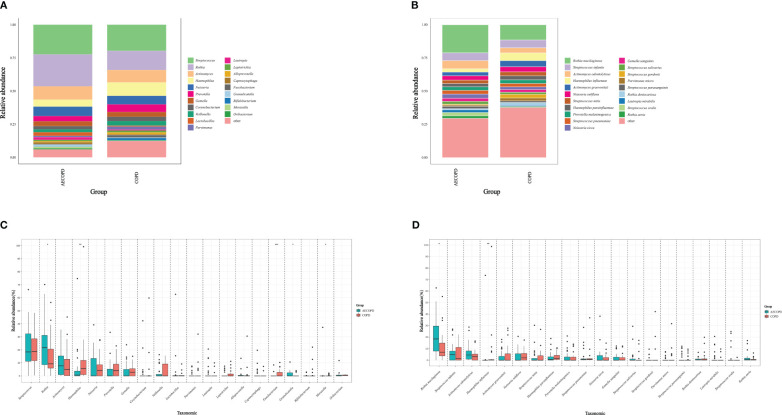
Microbiota compositional profiles and difference analysis between the AECOPD group and the COPD group. **(A, B)** Depicted the abundance of microbiota with bar charts in the genus level **(A)** and species level **(B)** respectively. The y-axis represents the percentage of each bacterium. The legend represents the color of each bacterium. **(C, D)** Depicted the distinguished microbiota with boxplots in the genus level **(C)** and species level **(D)** respectively, *p < 0.05, **p < 0.01, ***p < 0.001.

The microbiota compositional profiles were exhibited in **Figure 1**. In the genus level, *Fusobacterium*(accounts for 0.38% in the AECOPD group vs 1.78% in the COPD group, *p*<0.001) and *Haemophilus*(5.31% vs 10.11%, *p*=0.007) increased in the AECOPD group, *Moraxella*(1.20% vs 0.20%, *p*=0.039), *Rothia*(24.07% vs 14.43%, *p*=0.032) and *Granulicatella*(1.43% vs 0.40%, *p*=0.018) deceased in the AECOPD group. In the species level, *Rothia mucilaginosa* (21.07% in the AECOPD group vs 11.41% in the COPD group, *p*=0.015) increased and *Haemophilus influenzae* (2.41% vs 5.76%, *p*=0.015) decreased when compared the AECOPD group to the COPD group.

### 3.3 Differential expression analysis

**Figure 2:**
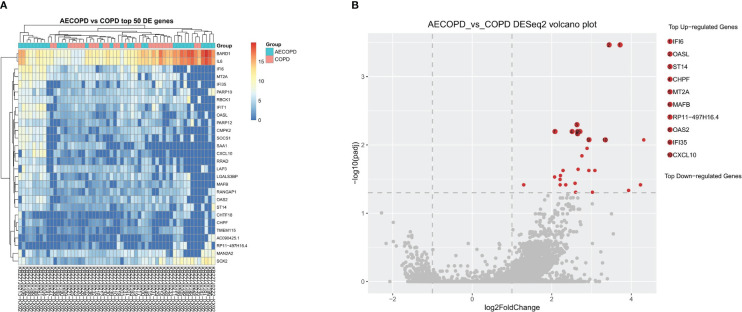
Differentially Expressed Genes between AECOPD and COPD patients. According to the relative abundance of DEGs in each sample, the Top 50 different DEGs were selected to draw a heatmap **(A)** between the AECOPD group and the COPD group. All 50 DEGs were upregulated. The color gradient and similarity degree reflect the similarity and difference of two samples at different classification levels, the warmer the tone, the greater the value. **(B)** Illustrated the volcano map of DEGs between AECOPD and COPD patients. Red dots, *p*-adjusted<0.05. Black dots, *p*<0.05. Bounded by Log2 Fold Change=0 on the abscissa, the left side represents down-regulated genes and the right side represents up-regulated genes.

After RNA sequencing and analysis, 2229 DEGs were selected, 2014 of them were upregulated in AECOPD and 215 of them were downregulated in AECOPD. After *p*-value adjustment, a total of 28 upregulated DEGs were identified in the AECOPD group (**Table 2**). The corresponding volcano plots and heatmaps are displayed in **Figure 2**. Among them, Interferon Alpha Inducible Protein 6 (*IFI6*) showed the largest expression difference (*q*<0.001), while Oligoadenylate synthase-like (*OASL*) (*q*<0.001) demonstrated the most significant variation between the two groups.

**Table 2:** Twenty-eight DEGs between the AECOPD group and the COPD group.

Gene ID	Gene name	Log2(FC)	*p*-value	*p*-adjust
ENSG00000126709.10	IFI6	3.703	0.000	0.000
ENSG00000135114.8	OASL	3.429	0.000	0.000
ENSG00000149418.6	ST14	2.620	0.000	0.005
ENSG00000250918.2	RP11–497H16.4	2.067	0.000	0.006
ENSG00000125148.6	MT2A	2.693	0.000	0.006
ENSG00000204103.2	MAFB	2.637	0.000	0.006
ENSG00000123989.9	CHPF	2.498	0.000	0.006
ENSG00000111335.8	OAS2	2.635	0.000	0.007
ENSG00000068079.3	IFI35	2.921	0.000	0.008
ENSG00000181449.2	SOX2	4.303	0.000	0.008
ENSG00000169245.4	CXCL10	3.332	0.000	0.008
ENSG00000126062.3	TMEM115	2.878	0.000	0.011
ENSG00000185745.8	IFIT1	2.749	0.000	0.014
ENSG00000108679.8	LGALS3BP	2.653	0.000	0.023
ENSG00000173432.6	SAA1	3.074	0.000	0.024
ENSG00000178685.9	PARP10	2.274	0.000	0.024
ENSG00000185338.4	SOCS1	2.923	0.000	0.024
ENSG00000196547.10	MAN2A2	2.209	0.000	0.028
ENSG00000125826.15	RBCK1	2.062	0.000	0.029
ENSG00000002549.8	LAP3	2.200	0.000	0.032
ENSG00000134326.7	CMPK2	2.576	0.000	0.037
ENSG00000138376.6	BARD1	4.221	0.000	0.038
ENSG00000100401.15	RANGAP1	2.201	0.000	0.038
ENSG00000059378.8	PARP12	2.342	0.000	0.038
ENSG00000213165.2	AC090425.1	1.285	0.000	0.038
ENSG00000136244.7	IL6	3.922	0.000	0.046
ENSG00000127586.12	CHTF18	3.013	0.000	0.049
ENSG00000166592.7	RRAD	2.591	0.000	0.049

**Figure 3:**
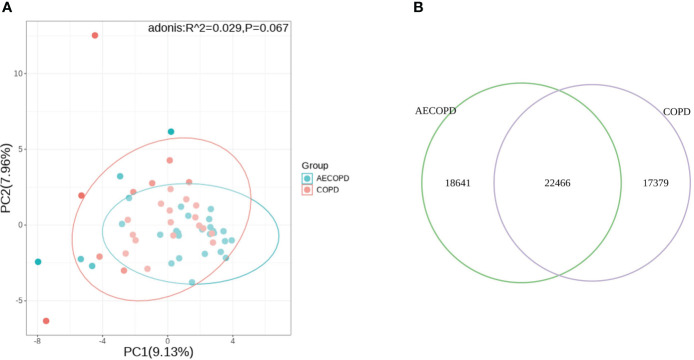
Comparison of microbial and transcriptional composition between groups. **(A)** PCA analysis of microbial data between groups. Microbiota composition bear no significant difference between the AECOPD group and the COPD group. **(B)** Venn map illustrating the abundance and similarities of different expressed genes.

Principal Component Analysis(PCA) analysis was carried to present the difference of microbiota structure between groups. The two groups were not significantly separated at PC1 and PC2 coordinates(*p*=0.067), considering that there was no significant difference in microflora deconstruction between the two groups (**Figure 3A**). The Venn diagram is used to visualize the number of DEGs in both groups. The two groups shared 22,466 genes, accounting for 54.7% of group AECOPD and 42.3% of group COPD (**Figure 3B**).

**Figure 4:**
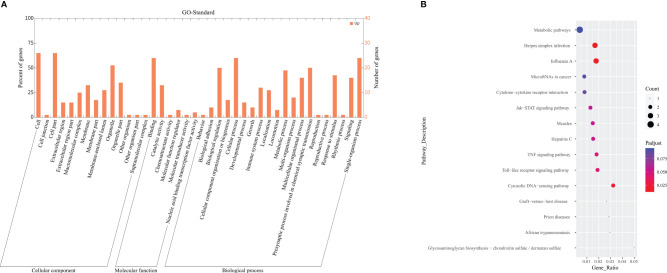
GO and KEGG enrichment analysis. **(A)** Depicted the GO term annotation of DEGs in a histogram. The x-axis represents the gene function in the GO standard. The y-axis represents the number of genes gathered, and the ratio of DEGs aggregated in this term. **(B)** Demonstrated a Plotdot portrayed accordingly according to the KEGG Pathway enrichment analysis of the AECOPD and COPD groups. The Y-axis represents the pathway of each molecule to play a function. The X-axis means Gene Ratio, which is the proportion of genes enriched to the target pathway gene in the gene list. Bubble area size means the number of enriched genes. Bubble color means enrichment significance, that is, the size of the *q* value.

Among the categories of "Cellular Component" (CC), "Molecular Function" (MF), and "Biological Process" (BP) in GO enrichment analysis, we observed significant enrichment of DEGs in "type I interferon signaling pathway" (*p*<0.001, *q*=0.001) and "defense response to virus" (*p*<0.001, *q*=0.023). The integrated gene annotation was presented in **Figure 4A**, from which we can observe the aggregation of DEGs in intracellular material production. KEGG Enrichment Analysis revealed five distinguished upregulated pathways. These pathways include "Influenza A" (*p*<0.001, *q*=0.012), "Herpes simplex infection" (p<0.001, *q*=0.014), "Cytosolic DNA-sensing pathway" (*p*=0.002, *q*=0.024), "Toll-like receptor signaling pathway" (*p*=0.006, *q*=0.045), and "TNF signaling pathway" (*p*=0.006, *q*=0.045), The plotdots representing these pathways are displayed in **Figure 4B**. Based on the aforementioned results, we produced **Table 3** to integrate key pathways and related genes.

**Table 3:** Key upregulated pathways and related genes between AECOPD group and COPD group.

Terms	Database	*p*-value	*p*-adjust	Regulate	Gene Name
type I interferon signaling pathway	GO	<0.001	0.001	UP	*IFI35|OAS2|IFI6|OASL|IFIT1*
defense response to virus	GO	<0.001	0.023	UP	*OAS2|OASL|IL6|CXCL10|IFIT1*
Influenza A	KEGG PATHWAY	0.001	0.012	UP	*IL6|CXCL10|OAS2*
Herpes simplex infection	KEGG PATHWAY	0.001	0.014	UP	*IFIT1|IL6|OAS2*
Cytosolic DNA-sensing pathway	KEGG PATHWAY	0.002	0.024	UP	*IL6|CXCL10*
Toll-like receptor signaling pathway	KEGG PATHWAY	0.006	0.045	UP	*IL6|CXCL10*
TNF signaling pathway	KEGG PATHWAY	0.006	0.045	UP	*IL6|CXCL10*

### 3.4 Relative quantification and comparison analysis of host genes

**Figure 5:**
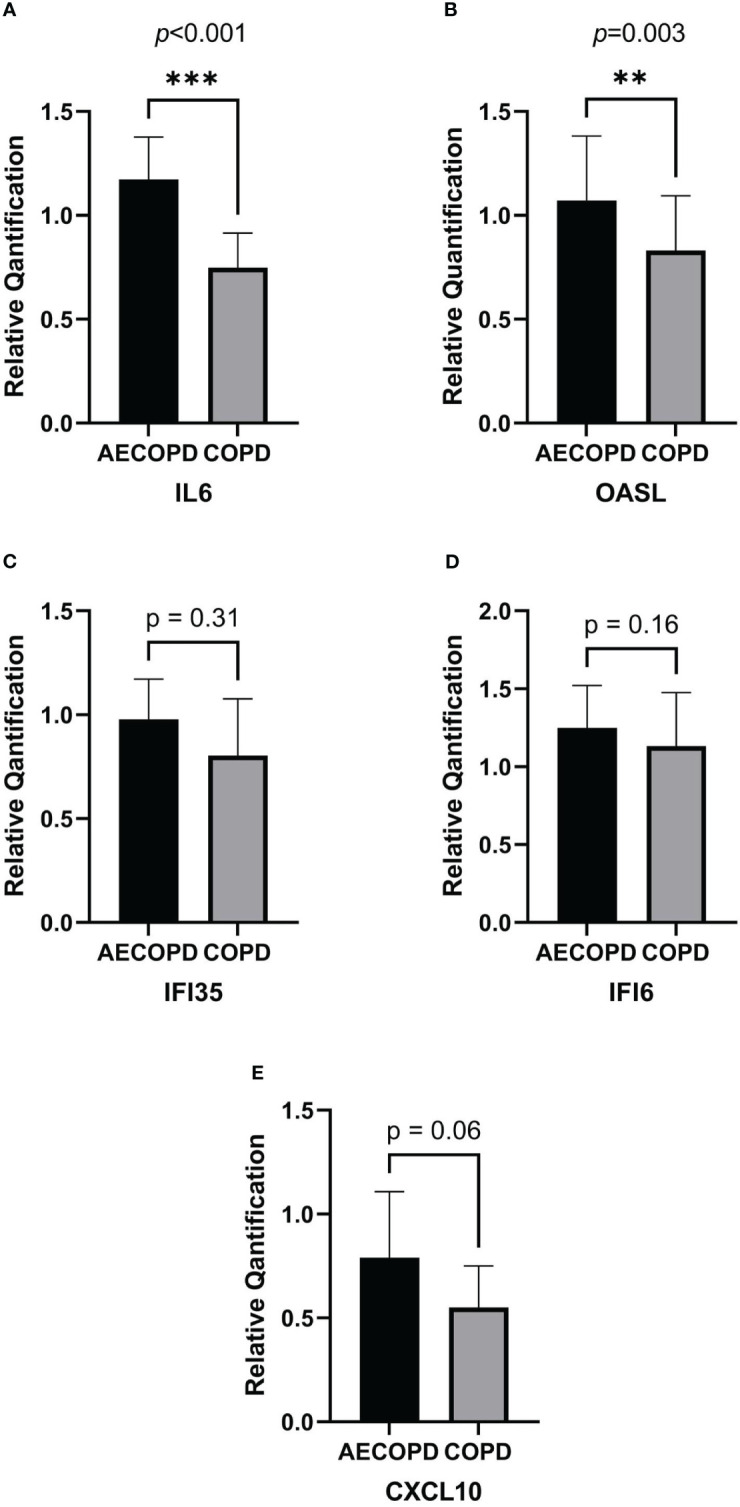
Relative quantification of DEGs by qPCR and gene expression comparison analysis. The horizontal coordinate of the column bar graph represents the grouping, and the vertical coordinate represents the relative quantitative data of each gene. Student's t test or non-parametric test were used for comparison between the two groups. ***p<0.001, **p<0.01. **(A)** IL6. **(B)** OASL. **(C)** IFI35. **(D)** IFI6. **(E)** CXCL10.

According to the RNA isolation results and DEG analysis, we used Quantitative Real-time PCR(qPCR) to quantify the gene expression of *OASL*, *IL6*, *IFI35*, *IFI6* and *CXCL10* in each sample. **Figure 5** was plot after gene quantification analysis and discriminant analysis. The expression of *IL6*(*p*<0.001) and *OASL*(*p*=0.003) were significantly elevated in the AECOPD group. *IFI35*(*p*=0.31), *IFI6*(*p*=0.16) and *CXCL10*(*p*=0.06) demonstrated no difference. Original data can be found in **Supplementary Material 2**.

### 3.5 Correlation analysis between DEGs and clinical markers

**Figure 6:**
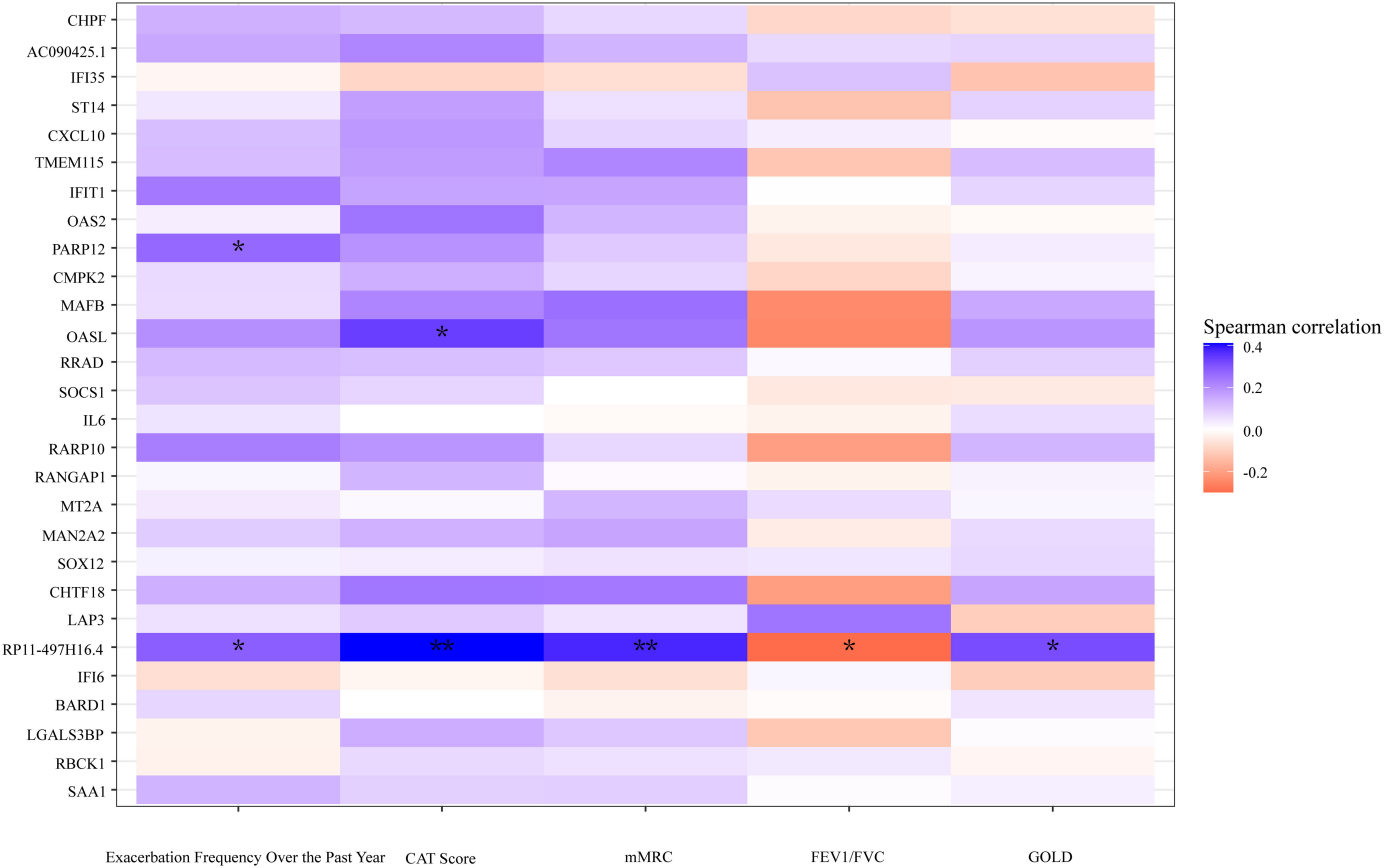
A heatmap plot according to the correlation of DEGs and clinical indicators. Cool tones represent a positive correlation and warm tones represent a negative correlation. The color gradient represents the degree of correlations, the darker the color, the higher the correlation. **p<0.01, *p<0.05, two tailed.

Spearman's rank correlation coefficient was calculated between related clinical indicators and DEGs, revealing that the pseudogene "*RP11–497H16.4*" exhibited a positive correlation with CAT (*r*=0.40, *p*<0.01), mMRC (*r*=0.37, *p*<0.01), and GOLD (*r*=0.32, *p*<0.05), as well as a negative correlation with FEV1/FVC (*r*=-0.32, *p*<0.05). Additionally, "*OASL*" had a positive correlation with CAT as well (*r*=0.34, *p*<0.05) (**Figure 6**). The original data can be found in **Supplementary Material 3**.

### 3.6 Microbiota network analysis and correlation with different expressed genes

**Figure 7:**
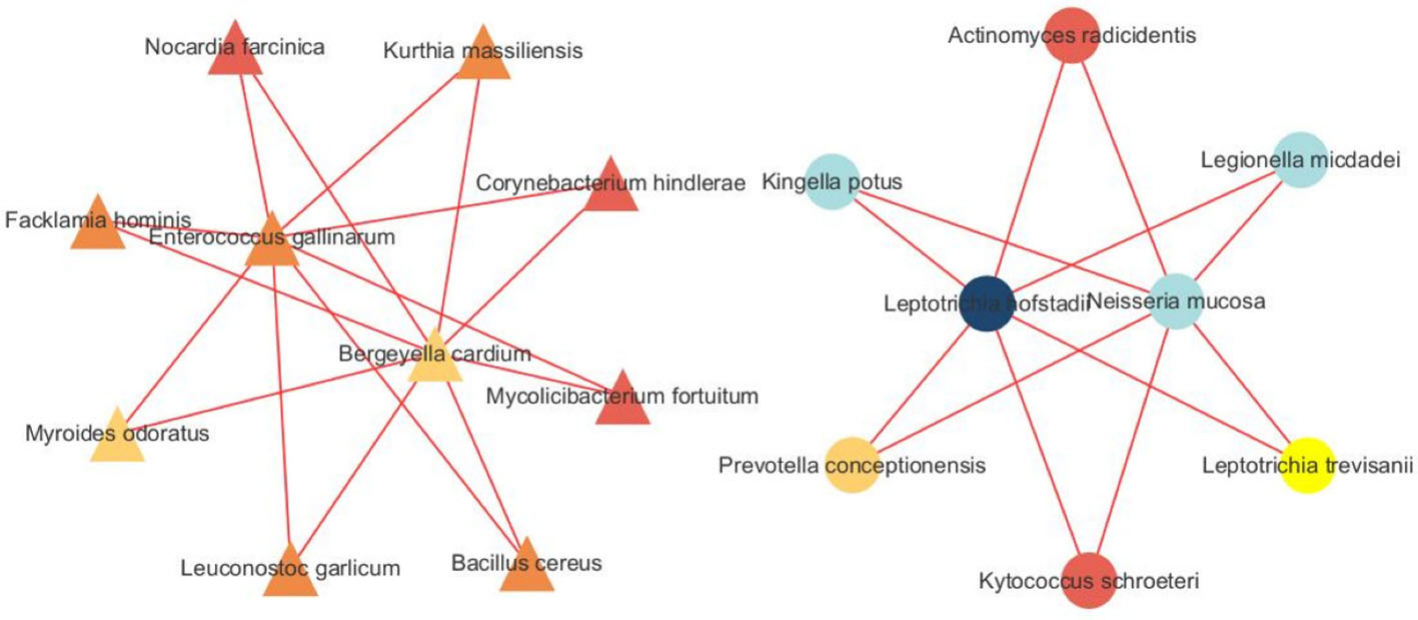
Microbiota network analysis. The triangle represents species originated form the SCOPD group, the circle represents species originated form the AECOPD group. The results show that different nodes represent different dominant species, and the connections between nodes indicate that there is a correlation between the two species. The stronger the correlation, the obvious connection lines, where the red line indicates a positive correlation and the blue line indicates a negative correlation. In addition, node colors are defined by gate level, and node colors are the same, indicating that these species belong to the same gate. Through the number of node connections, it is possible to identify species that are more related to other members of the flora, and then explore the biological significance of the correlation between these species.

**Figure 8:**
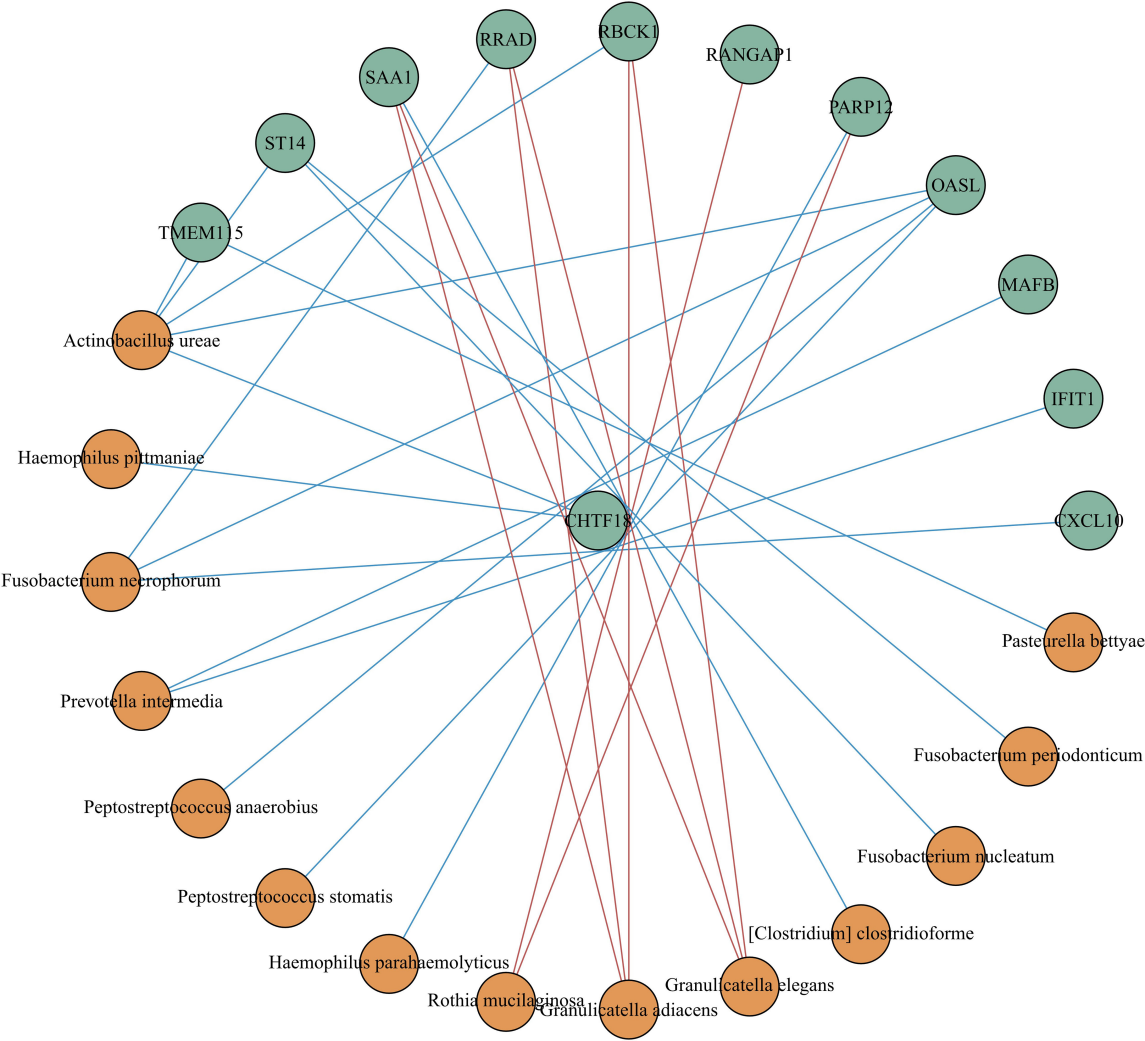
Correlation analysis between different expressed genes and microbiota. Spearman correlation analysis was performed using the relative abundance of all bacteria at the species level and the DEGs. The green circle represents the DEGs, the orange circle represents the microbiota. The red line means positive correlation, and the blue line means negative correlation.

We built a correlation network in the species level in **Figure 7**. *Entercoccus gallinarum*(*Bacillota* phylum) and *Bergeyella cardium*(*Bacteroidota* phylum) were recognized as the core microbiome of the COPD group. Leptotrichia hofstadii(Fusobacteriota phylum) and Neisseria mucosa(Pseudomonadota phylum) were the most associated microbiome in the AECOPD group. We correlated the microbial data with the DEGs in **Figure 8**. The results exhibited a positive correlation in the abundance of *Rothia mucilaginosa* with *RANGAP1* and *PARP12* expression, it manifested the negative correlation in the abundance of *OASL* expression with *Actinobacillus ureae*, *Fusobacterium necrophorum*, *Peptostreptococcus anaerobius*, and *Peptostreptococcus stomatis* as well.

## 4 Discussion

Previous study showed that *Proteobacteriato*-*Firmicutes* ratio and *Prevotella* were found to be easily influenced by Inhaled Corticosteroids (ICS) use (Ramsheh et al., 2021), and smoking generally resulted in an elevated abundance of *Haemophilus (* Hunt et al, 2020). To eliminate these interferences, we controlled the ICS use and smoking history with no statistic difference while recruitment. In our study, we presented microbial composition and host transcriptional profiles of 31 patients underwent exacerbation and 26 patients at stable stage. This is the first study to discuss the potential mechanism underlying the exacerbation of COPD, as far as we know.

The microbial compositional analysis elucidated a higher expression of *Fusobacterium* and *Haemophilus* in the stable stage COPD, and *Rothia*, *Moraxella*, and *Granulicatella* were more prevalent during acute exacerbation. The transcriptional profile indicated an integration of DEGs in type I interferon (IFN) signaling pathway and TLRs signaling pathway.

Type I IFNs mainly include IFN-α and IFN-β, which are important effector molecules involved in antiviral immunity. Bacterial infection induces type I IFNs as well (Gonzalez-Navajas et al., 2012). Its production is mainly induced by surface or internal receptors (such as TLRs and cGAS) on innate immune cells (mainly macrophages) upon their contact with virus-specific antigenic substances (DNA, RNA). Then it is transmitted by intracellular signaling molecules, and activated transcription factor IRF3/7 to initiate the expression of type I IFN genes. This pathway promotes the pathogenesis of COPD via miR-21/SATB1/S100A9/NF-κB axis (Kim et al., 2021).

Toll-like receptors (TLRs) are associated with microbiome-COPD interplay. TLRs are momentous mediators for pathogens to recognize exogenous pathogen-associated molecular patterns (PAMPs) and host-derived damage-associated molecular patterns (DAMPs), inducing and sustaining the inflammation caused by the microbiome in COPD selectively (Zuo et al., 2015). TLR4 acted as a pathogen recognition receptor to identify lipopolysaccharides (LPS) or endotoxins produced by gram-negative bacteria (Knobloch et al., 2011; Oliveira and Reygaert, 2023), subsequently initiates the TIR-domain-containing adapter-inducing interferon-β signaling pathway and upregulated the expression of type I IFN (De Nardo, 2015; Zuo et al., 2015; Gajanayaka et al., 2021). *Haemophilus*, *Moraxella*, and *Fusobacterium* all functioned in this way to some extent.

Through gene function annotation, we discovered that genes associated with type I IFN signaling pathways, such as *IFI6*, *IFI35*, *OASL*, *OAS2* and *IFIT1*, were integrated in the AECOPD group. Among them, *OASL* was verified to upregulated in AECOPD group by qPCR experiment using the same sample. *OASL* is a crucial factor in the activation of type I IFNs and it was acknowledged as the product induced by virus infection, but many studies revealed the elevated expression of *OASL* during bacterial infection (Kutsch et al., 2008; Weiss et al., 2010). However, the mechanism of his role in bacterial infection is not clear, and scholars speculate that it may be consistent with the pathway of viral infection (Leisching et al., 2017), which is associated with the phagocytosis of macrophages. *OAS2* is part of OAS family, its expression was relatively low in this study. *IFI6* has been reported to exhibit antiviral activity toward the *Hepatitis C Virus* (HCV) (Liu et al., 2019) and the *Influenza A Virus*. *IFI35* negatively regulates NF-κB when complexed with an N-Mye interactor (Jian et al., 2018), thus, acting negatively toward the development of COPD. Additionally, *IFI35* can also promote inflammation via activating macrophages through DAMPs, making it a potential treatment therapy for COPD.

Through KEGG pathway enrichment analysis, *IL6* and *CXCL10* expressed higher and were integrated in pathways concerning TLRs, TNFs, and Cytosolic DNA-sensing. qPCR amplification experiment validated that the expression of *IL6* resembled significant increasement in the AECOPD group than the stable COPD group. Previous study suggested the negative correlation between *IL6* expression and pulmonary function (Zhu et al., 2022). Besides, *IL6* was related to bacterial infection and inflammation response (Tang et al., 2012; Zhu et al., 2022), correlated *IL6ST* was reported to elevate when COPD aggravates (Ko et al., 1988). However, no specific study in relation to COPD pathogenesis and *IL6* expression has been done so far. It needs to be further explored for the intervention value. *CXCL10* is involved in a wide variety of processes during pathogen invasion and has been reported to modulate an individual's susceptibility to COPD (Wang et al., 2018). Inhibited *CXCL10* protects against COPD progression by reducing the secretion of inflammatory factors (Jing et al., 2018; Ju and He, 2021). Therefore, inhibiting type I IFNs and upstream inflammatory factors could potentially postpone COPD progression. We also found a certain positive relationship between *OAS2*, *CXCL10*, and herpes simplex infection.

This study identified several other host genes. *CHPF* encodes a protein that mainly functions in the chondroitin sulfate biosynthetic process, which is strongly correlated with matrix metalloproteinases, a proteinase that contributes to airway remodeling in COPD (Papakonstantinou et al., 2016). Studies have shown that *MAFB* participation in the pathophysiology of COPD affects the maturation and differentiation of macrophages and generates MMPs, ultimately leading to aggravated pulmonary emphysema and airflow restriction (Sato et al., 2011; Aida et al., 2014). The protein encoded by *ST14* is an epithelial-derived, integral membrane serine protease, while *MT2A* is a member of the metallothionein family and acts as an antioxidant *in vivo*. No research related to COPD and these genes has been carried out so far. Genes detected by microbiome-host analysis could have the potential to screen those who were susceptible to pathogen infections and intervene at an early stage.

Our research identified an unprocessed pseudogene, *RP11–497H16.4*, which was remarkably elevated and strongly associated with many clinical assessment indicators. This 1180 bp length gene has no protein transcripted and possibly originated from sequence changes after gene duplication.

*H.influenzae*, which belongs to the *phyla proteobacteria*, has been reported to act as an opportunistic pathogen by inducing a neutrophilic-mediated inflammation via the activation of PAMPs and the inflammatory cascade that follows (Bafadhel et al., 2015; Barker et al., 2015; Brightling and Greening, 2019). This process recruited both innate and adaptive immune cells, like macrophages, endothelial and epithelial cells (Sidletskaya et al., 2020). Furthermore, CD40, CD83, and CD86 expressed in monocyte-derived cells have also been found to be involved (Larsen et al., 2012; Oliveira and Reygaert, 2023). *Haemophilus* was commonly considered to be one of the initiating pathogens of type I IFNs (Lu et al., 2018; Yang et al., 2019) via TLRs. *Moraxella* was acknowledged as an inducement (Ramsheh et al., 2021) and a major factor (Wang et al., 2019) of AECOPD. As a gram-negative bacteria, it can induce TLR2-initiated inflammatory responses generated by pulmonary epithelial cells (Slevogt et al., 2008). Our research also found elevated levels of *Moraxella* in the AECOPD group. However, previous scholars were prone to a negative linear relationship between the expression level of type I IFNs production and the abundance of airway microbiota. Yoshihiko et al (Raita and Eur, 2022). deduced that a higher proportion of *Haemophilus* colonized in the nasopharynx was closely related to downregulated type I IFNs expression, which is consistent with our research observation. Wang Z et al (Wang et al., 2019). recognized *Haemophilus* as a stable stage colonizer as well. *Haemophilus* was thought to be an inducement of AECOPD before, now we think comprehensive research is needed in the future.

Elimination of type I IFN signaling was also found to improve clearance and survival following secondary bacterial pneumonia (Tian et al., 2012). However, Klaile E et al (Klaile et al., 2013). stressed that type I IFN could reduce the inflammation responses by enhancing the expression of The Carcinoembryonic Antigen-related Cell Adhesion Molecules (*CEACAM*) 1 due to negative interactions between *Moraxella* and *CEACAM1*. These research findings align with the hypothesis that type I IFNs are protective in acute viral infections but can have either protective or deleterious roles in bacterial infections (Trinchieri, 2010). We suppose that the viral-bacterial co-infection via type I IFNs could exhibit a competitive relationship, and it often occurs in the early stage of exacerbation in COPD. Once the damage has been caused by a virus infection, it tends to evolve into a Synergistic relationship. Unfortunately, our study did not analyze the viral load of research samples. However, through KEGG enrichment analysis, it is not difficult to find that microbe-host differential genes were enriched in viral infection pathways such as *influenza A Virus* and *Herpes Simplex Virus*.

The co-infection of viruses and bacteria has been acknowledged as a synergistic effect that correlates with the severity of COPD (Wilkinson et al., 2006; George et al., 2014). Preceding or concurrent viral respiratory tract infection can predispose to secondary bacterial co-infection via damaging the airway and dysregulating immune responses (Bakaletz, 2017). These infections can also induce neutrophil elastase, which can cleave the antimicrobial peptides *SLPI* and elafin (Mallia et al., 2012).

*R.mucilaginosa* is a normal flora of the oropharynx and is often detected in induced sputa samples. It has been reported to have an inhibitory effect on pathogen- or lipopolysaccharide-induced pro-inflammatory response via inhibiting NF-κB pathway activation and negatively correlated with pro-inflammatory markers (IL-8, IL-1β) and MMPs (Rigauts et al., 2022; Melo-Dias et al., 2023). Additionally, *Rothia* triggered a Th17 immune response and reduced the frequency of exacerbation in COPD patients as well (Ren et al., 2018). *Rothia* was observed to be significantly elevated in the AECOPD group but reduced in the stable COPD group in our research. Based on the anti-inflammatory effect of *Rothia* and our results, we suppose that *Rothia* may increase the bacterial load and play an anti-inflammatory role in the acute exacerbation of COPD through some potential mechanisms that need to be further explored. Therefore, *Rothia* may present itself as a potential biologic therapy to intervene in stable COPD patients, thus reducing the number of acute exacerbations and improving long-term prognosis.

Microbiota network analysis identified a few species that play central roles in microbial interactions, yet the abundance detected was low, and no statistical significance was observed after calculation as well. A multi-center or longitudinal analysis would be more suitable under this situation.

However, some drawbacks to our experiment must be acknowledged. Firstly, ICS can affect respiratory microflora over a long period. Although we controlled for no statistical difference in the history of ICS use between AECOPD and stable COPD, the type and dorse of ICS still need to be discussed. Secondly, although we obtained a rough estimate of viral load in our samples, further analysis relating to the virus load could refine our study. Thirdly, though the participants were all smokers, but the smoking index needs to stratify to explore the influence concerning smoking degree or smoking status(current or past).

## Data availability statement

The original data are available in NLM (nih.gov): PRJNA1022832.

## Ethics statement

The studies involving humans were approved by Huaian First People's Hospital: YX-2021-098-01. The studies were conducted in accordance with the local legislation and institutional requirements. The participants provided their written informed consent to participate in this study.

## Author contributions

YL: Formal analysis, Methodology, Project administration, Writing – original draft. XM: Data curation, Methodology, Writing – review & editing. PS: Data curation, Writing – review & editing. ZW: Resources, Writing – review & editing. DY: Investigation, Writing – review & editing. TM: Data curation, Writing – review & editing. BW: Visualization, Writing – review & editing. JPW: Software, Writing – review & editing. JJW: Funding acquisition, Writing – review & editing. RZ: Conceptualization, Supervision, Writing – review & editing.

## Supplementary material

Supplementary Material 1 Original data of recruiters

Supplementary Material 2 Original data of qPCR, including amplification curve and dissolution curve

Supplementary Material 3 Correlation analysis between DEGs and clinical markers
