# Supplementary material for: Microbiome-host interactions in the pathogenesis of acute exacerbation of chronic obstructive pulmonary disease
Source: Front Cell Infect Microbiol. 2024 Jul 18;14:1386201. doi: 10.3389/fcimb.2024.1386201 (PMC11291260; doi:10.3389/fcimb.2024.1386201)
Supplement: Supplementary Material 1 — Original data of recruiters [file DataSheet_1.pdf]

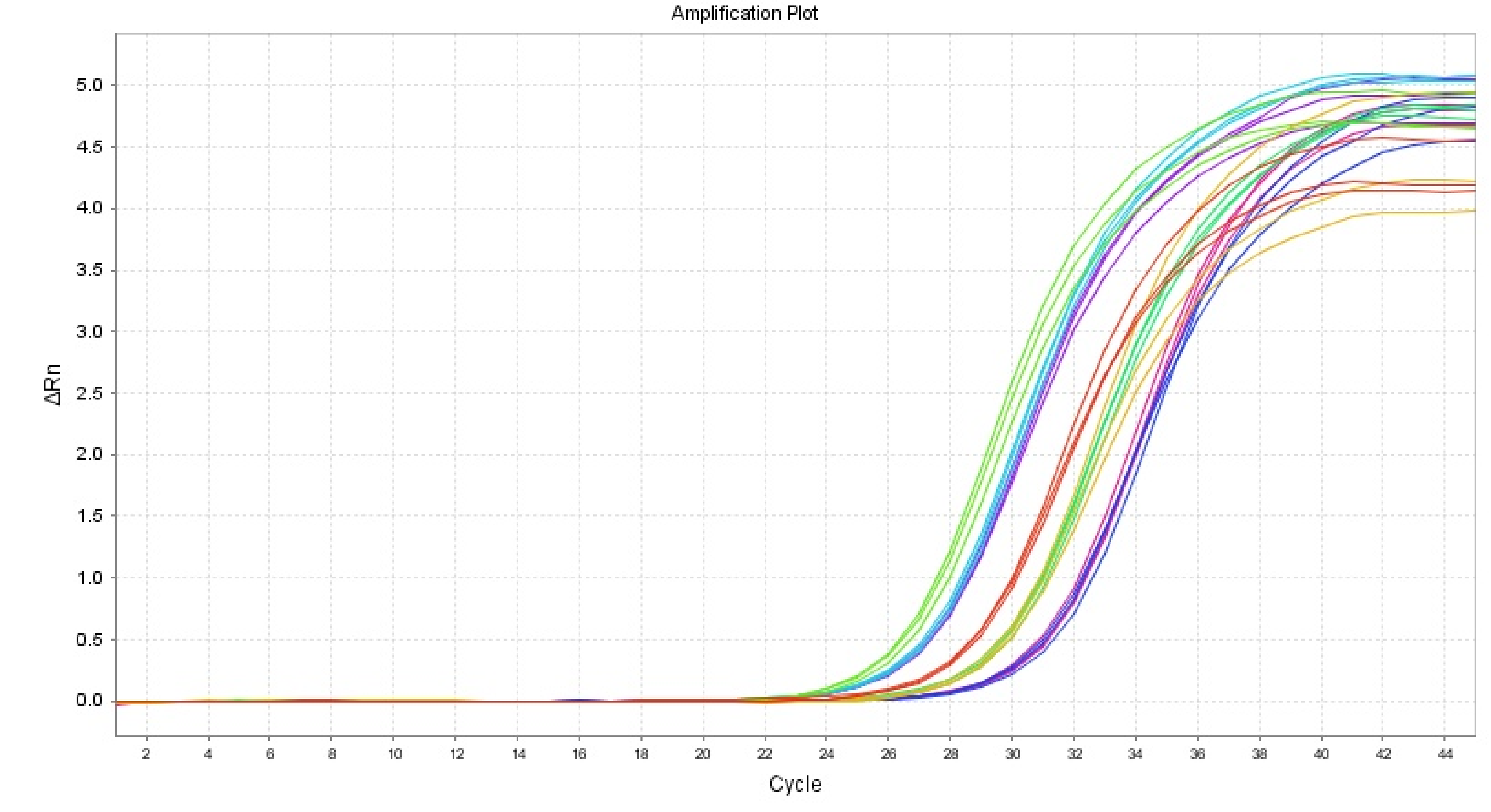
Amplification Plot
ΔRn
Cycle
0.0
0.5
1.0
1.5
2.0
2.5
3.0
3.5
4.0
4.5
5.0
2
4
6
8
10
12
14
16
18
20
22
24
26
28
30
32
34
36
38
40
42
44

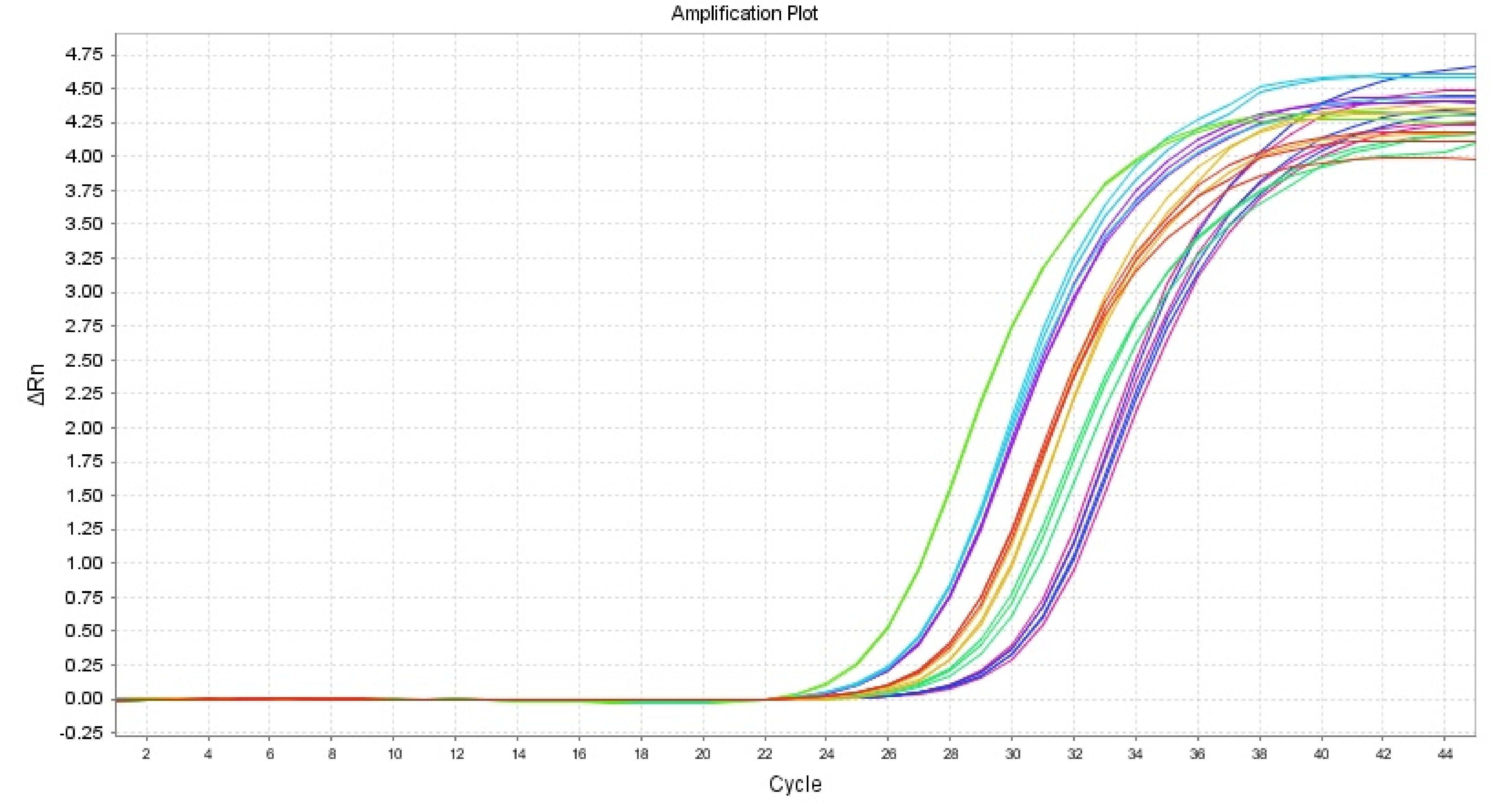
Amplification Plot
ΔRn
4.75
4.50
4.25
4.00
3.75
3.50
3.25
3.00
2.75
2.50
2.25
2.00
1.75
1.50
1.25
1.00
0.75
0.50
0.25
0.00
-0.25
2
4
6
8
10
12
14
16
18
20
22
24
26
28
30
32
34
36
38
40
42
44
Cycle

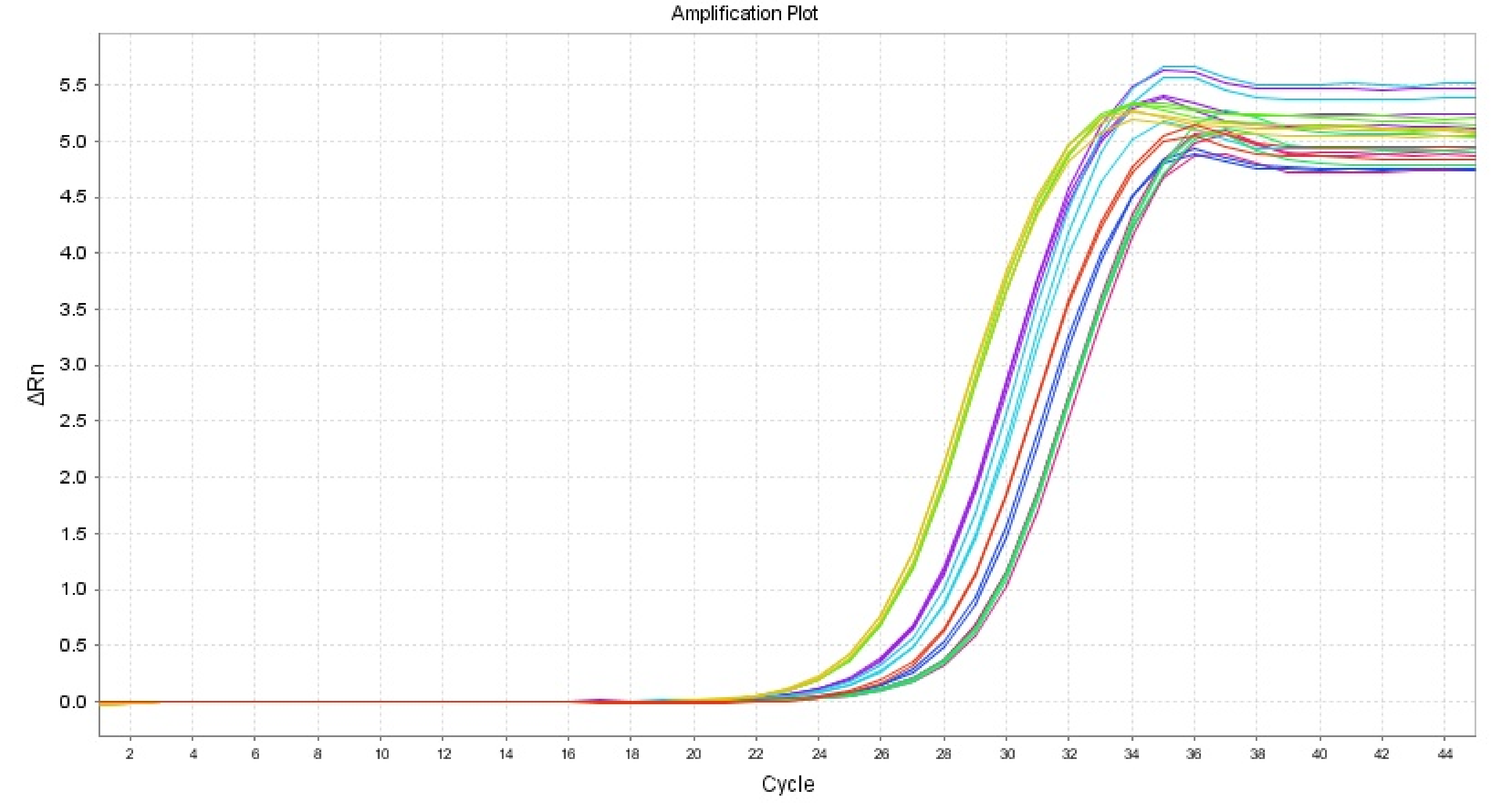

Amplification Plot
ΔRn
Cycle
0.0
0.5
1.0
1.5
2.0
2.5
3.0
3.5
4.0
4.5
5.0
5.5
2
4
6
8
10
12
14
16
18
20
22
24
26
28
30
32
34
36
38
40
42
44

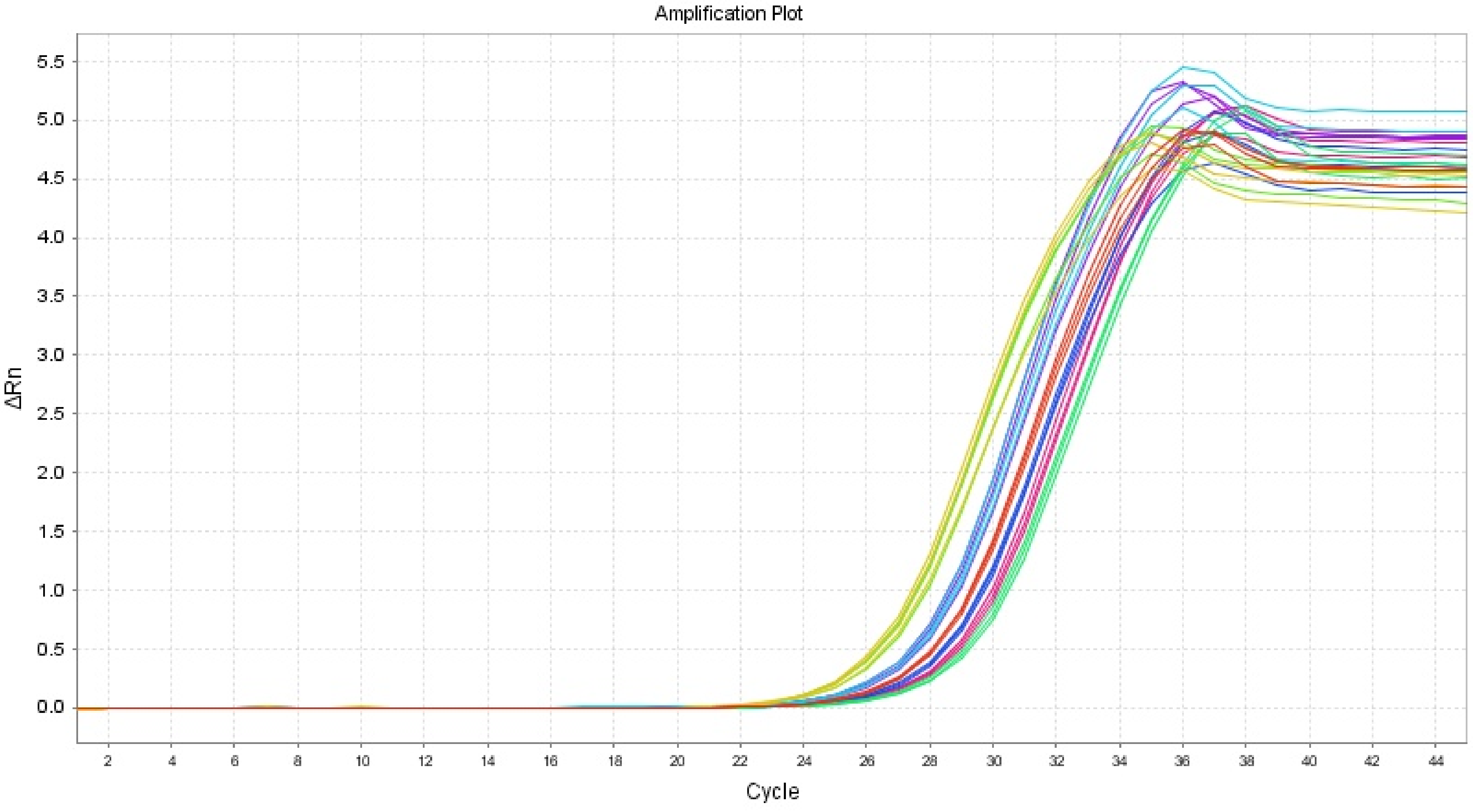
Amplification Plot
ΔRn
Cycle
5.5
5.0
4.5
4.0
3.5
3.0
2.5
2.0
1.5
1.0
0.5
0.0
2
4
6
8
10
12
14
16
18
20
22
24
26
28
30
32
34
36
38
40
42
44

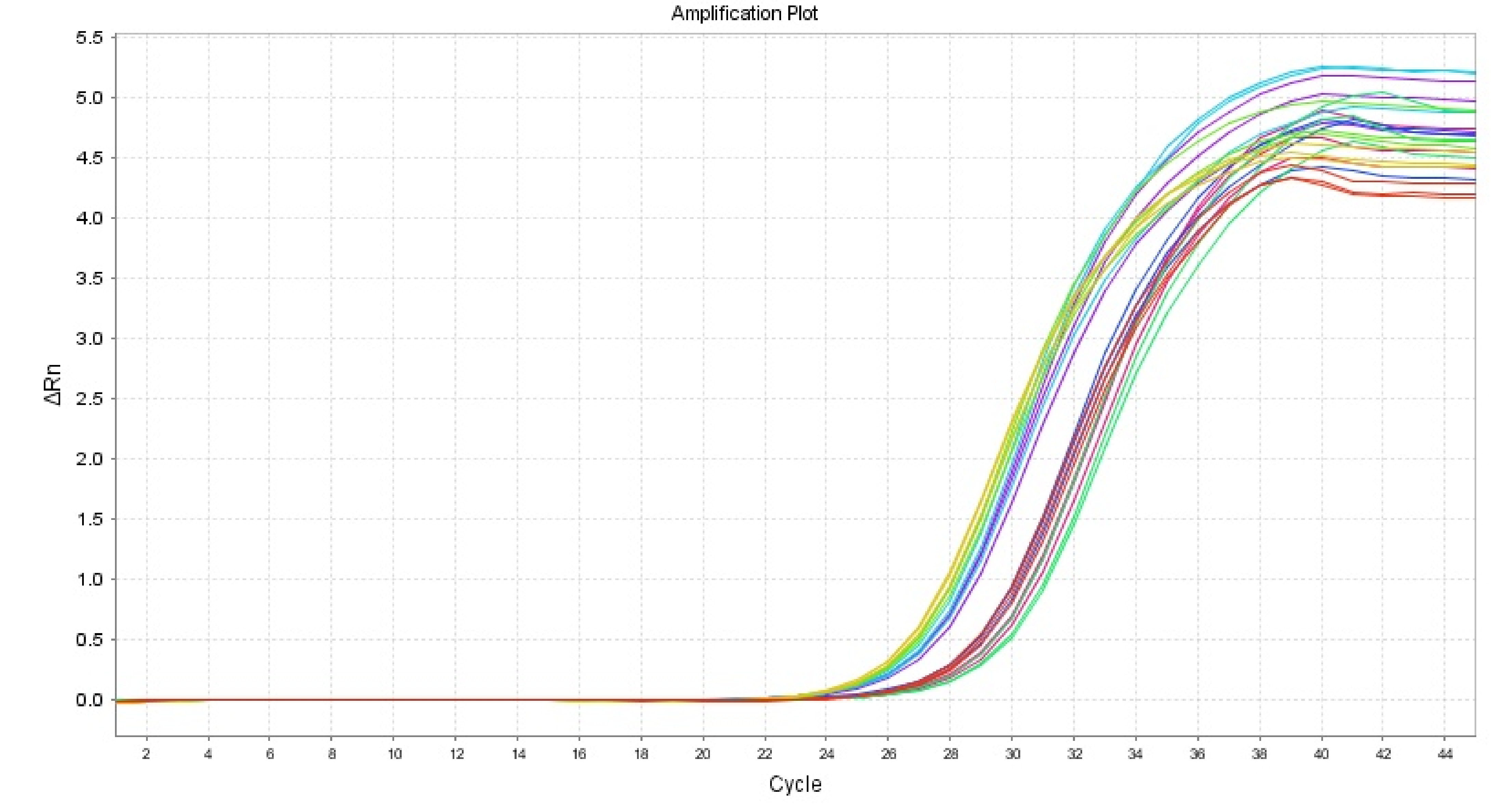

Amplification Plot
5.5
5.0
4.5
4.0
3.5
3.0
2.5
2.0
1.5
1.0
0.5
0.0
ΔRn
2
4
6
8
10
12
14
16
18
20
22
24
26
28
30
32
34
36
38
40
42
44
Cycle

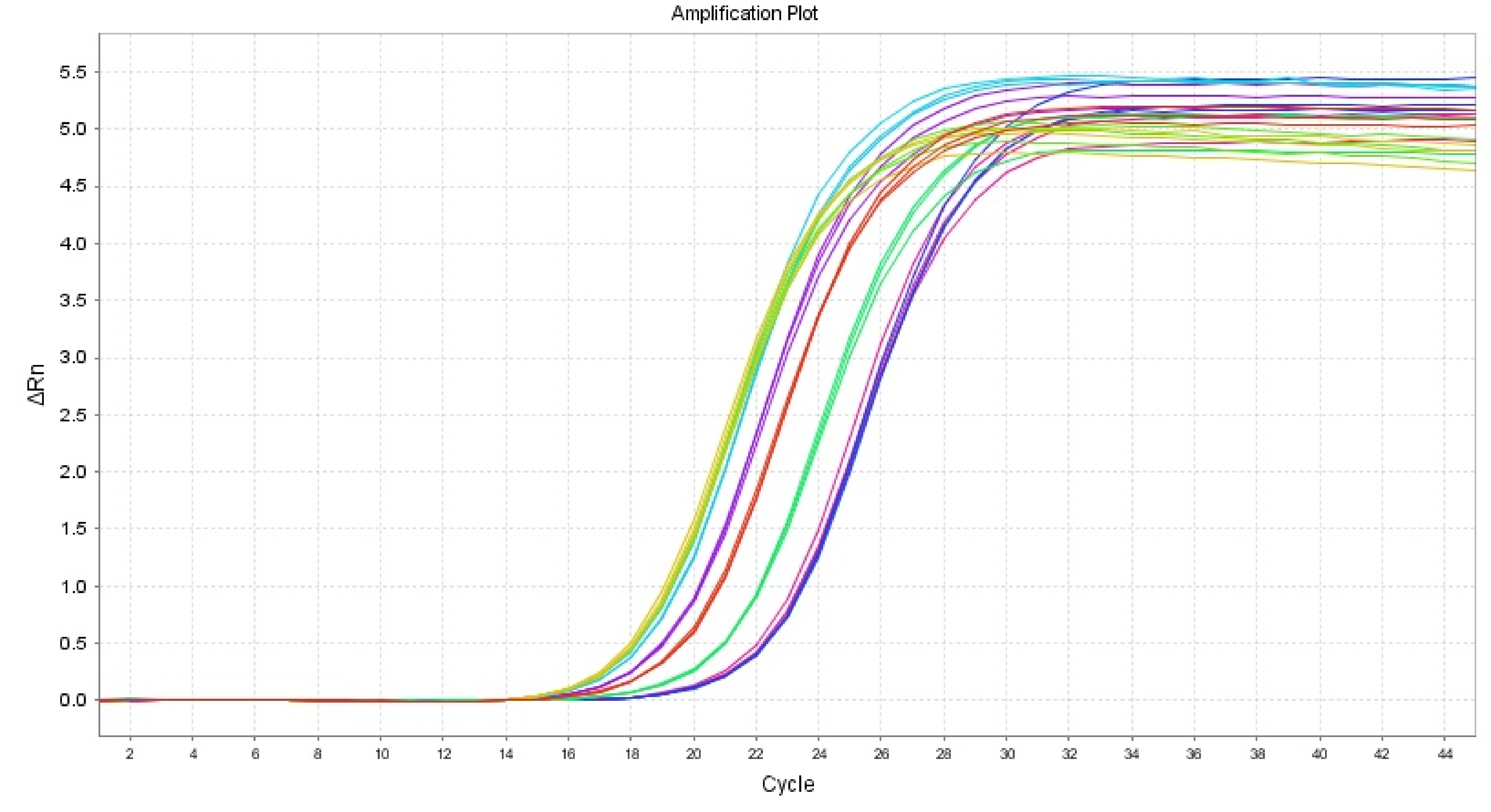
Amplification Plot
ΔRn
5.5
5.0
4.5
4.0
3.5
3.0
2.5
2.0
1.5
1.0
0.5
0.0
2
4
6
8
10
12
14
16
18
20
22
24
26
28
30
32
34
36
38
40
42
44
Cycle

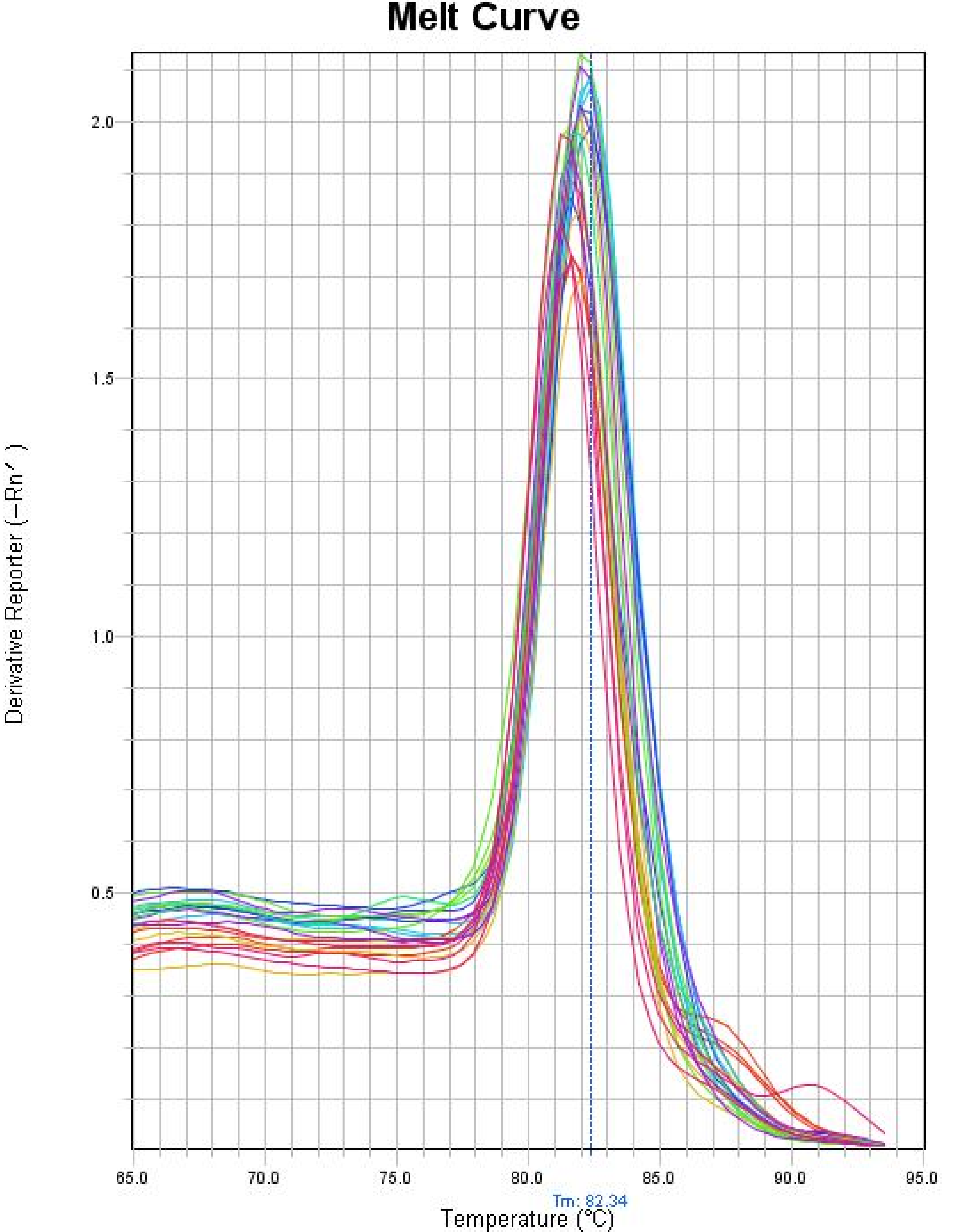

Melt Curve
Derivative Reporter (−Rn′)
2.0
1.5
1.0
0.5
65.0
70.0
75.0
80.0
85.0
90.0
95.0
Tm: 82.34
Temperature (°C)

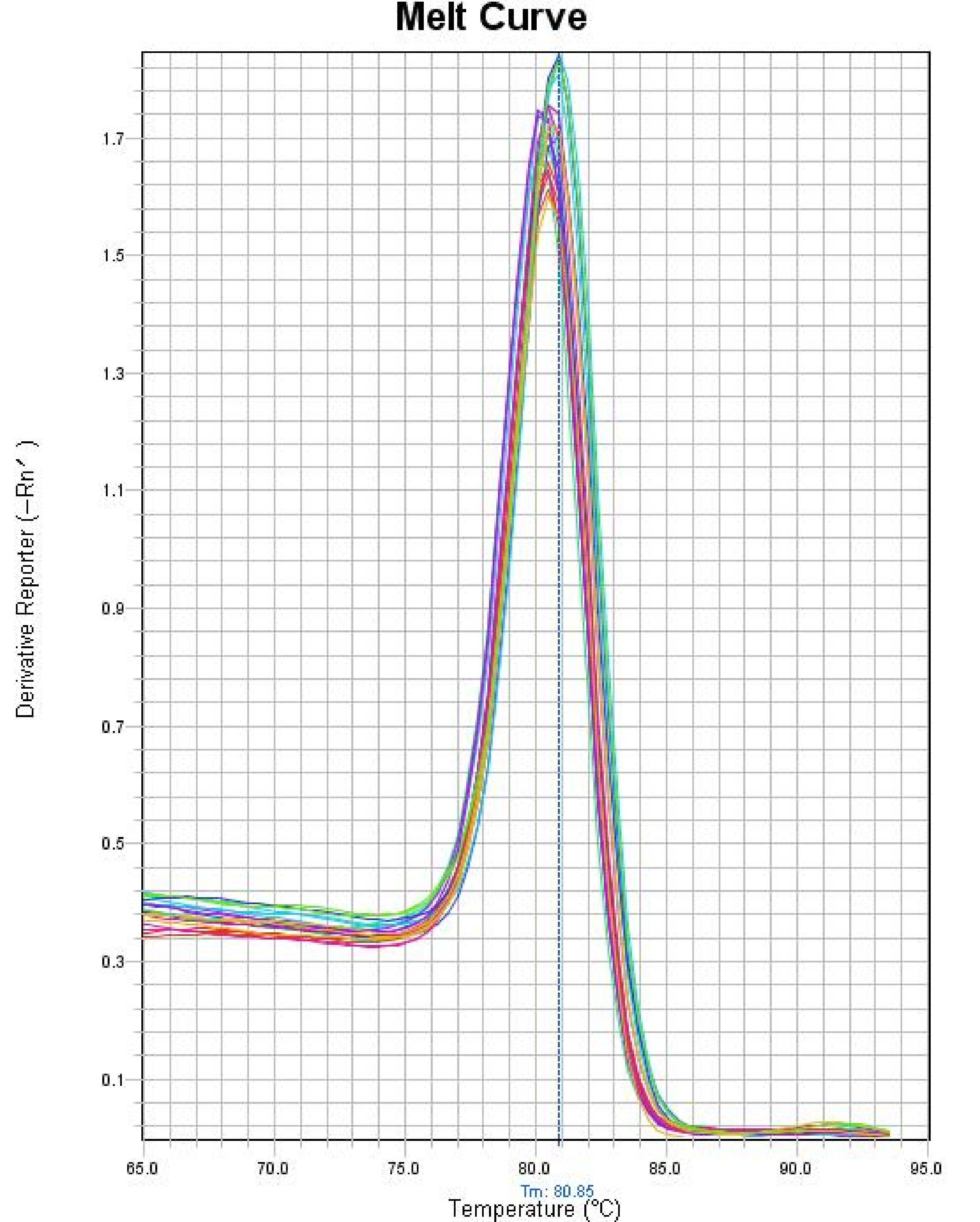

Melt Curve
Derivative Reporter (−Rn′)
1.7
1.5
1.3
1.1
0.9
0.7
0.5
0.3
0.1
65.0
70.0
75.0
80.0
85.0
90.0
95.0
Tm: 80.85
Temperature (°C)

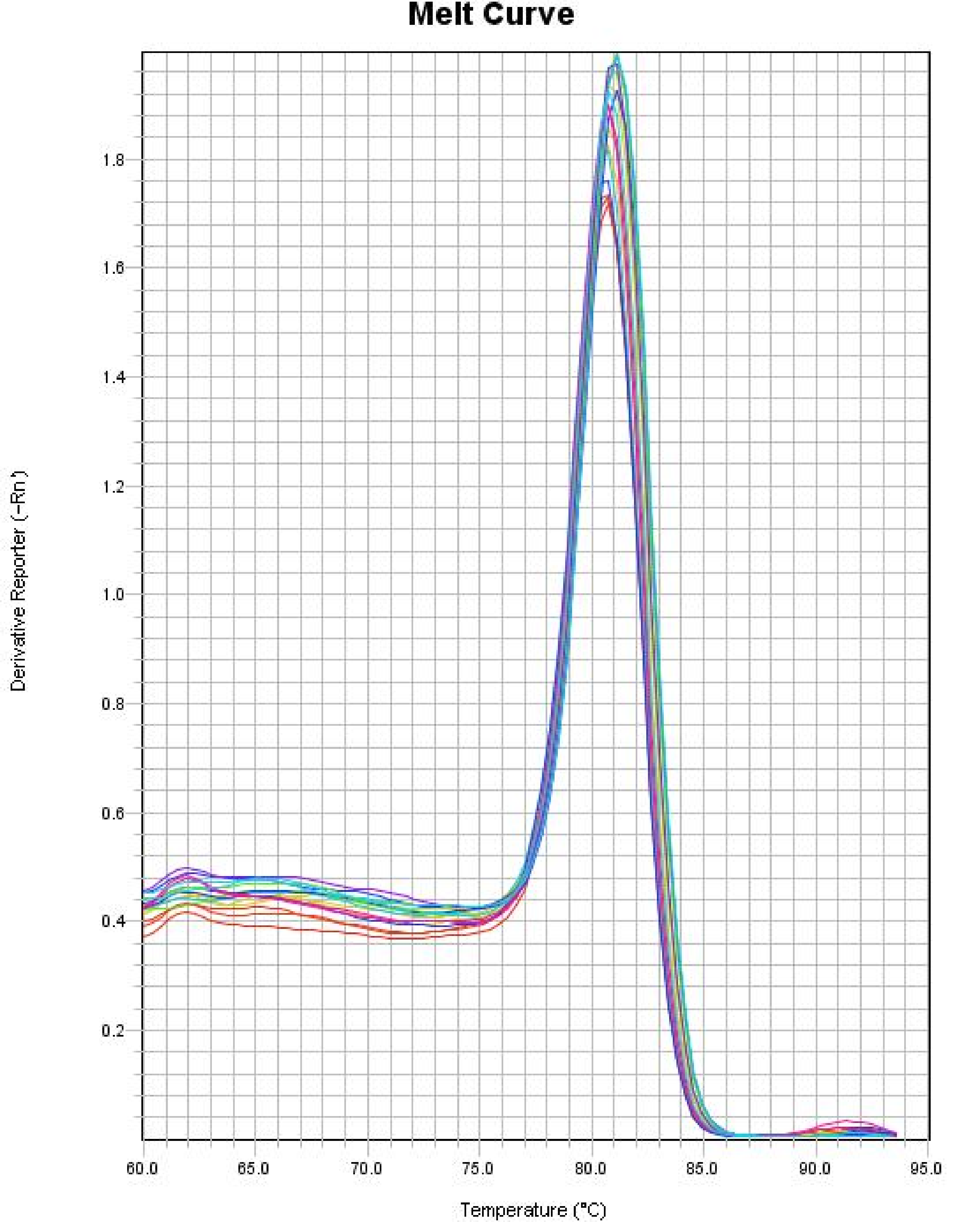
Melt Curve
Derivative Reporter (-Rn)
1.8
1.6
1.4
1.2
1.0
0.8
0.6
0.4
0.2
60.0
65.0
70.0
75.0
80.0
85.0
90.0
95.0
Temperature (°C)

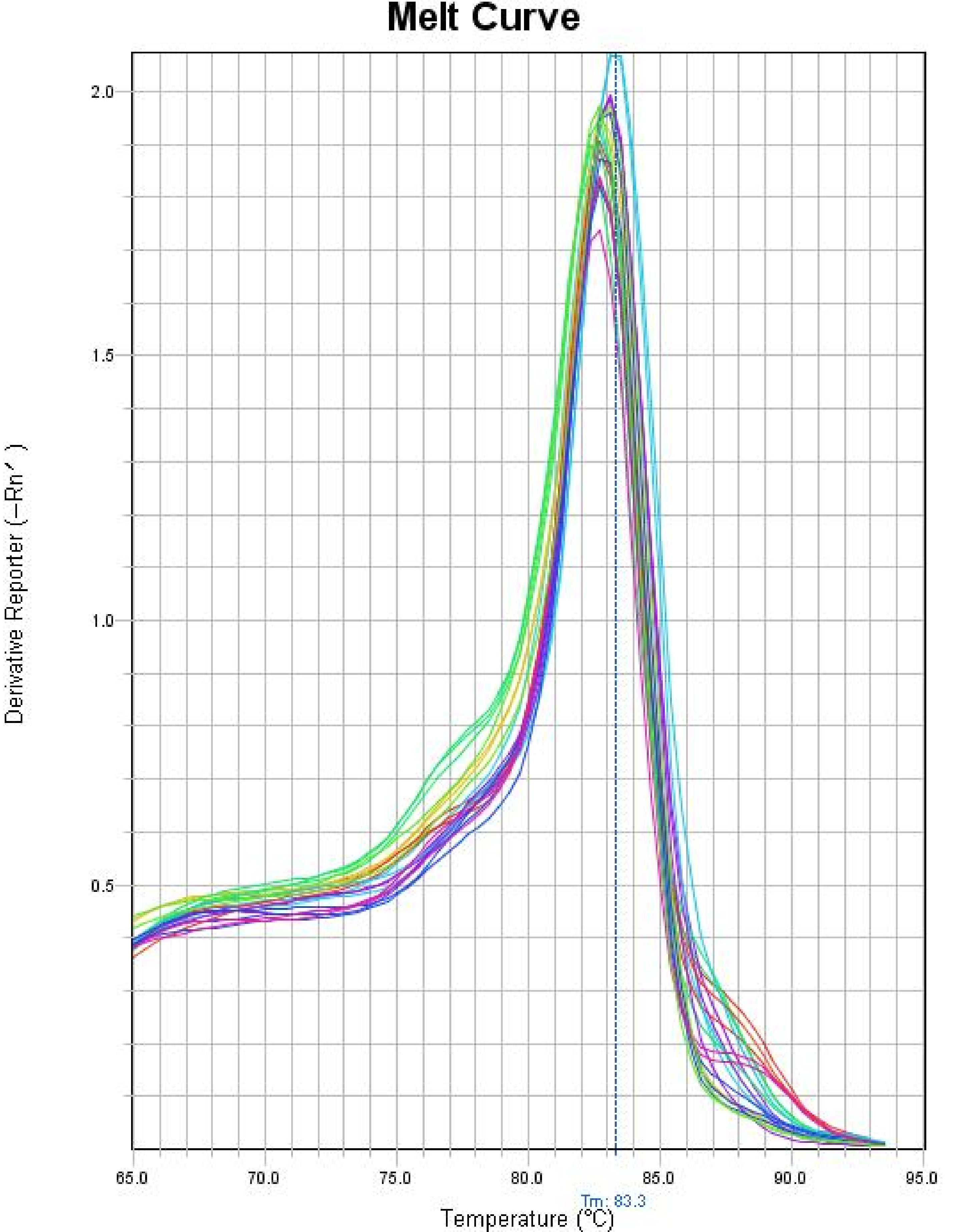
Melt Curve
Derivative Reporter (−Rn′)
2.0
1.5
1.0
0.5
65.0
70.0
75.0
80.0
85.0
90.0
95.0
Tm: 83.3
Temperature (°C)

Melt Curve

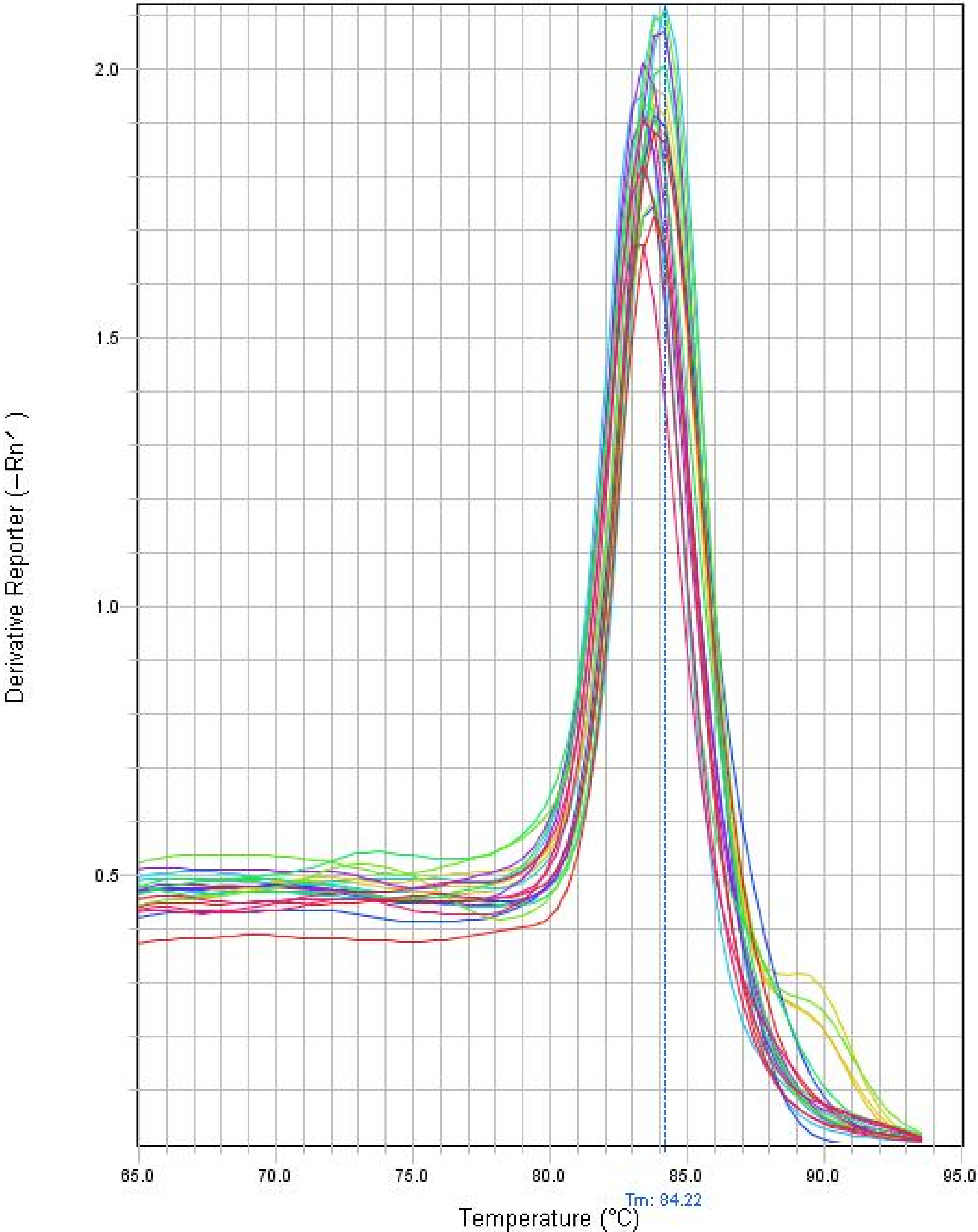

Derivative Reporter (−Rn′)
2.0
1.5
1.0
0.5
65.0
70.0
75.0
80.0
85.0
90.0
95.0
Tm: 84.22
Temperature (°C)

Melt Curve

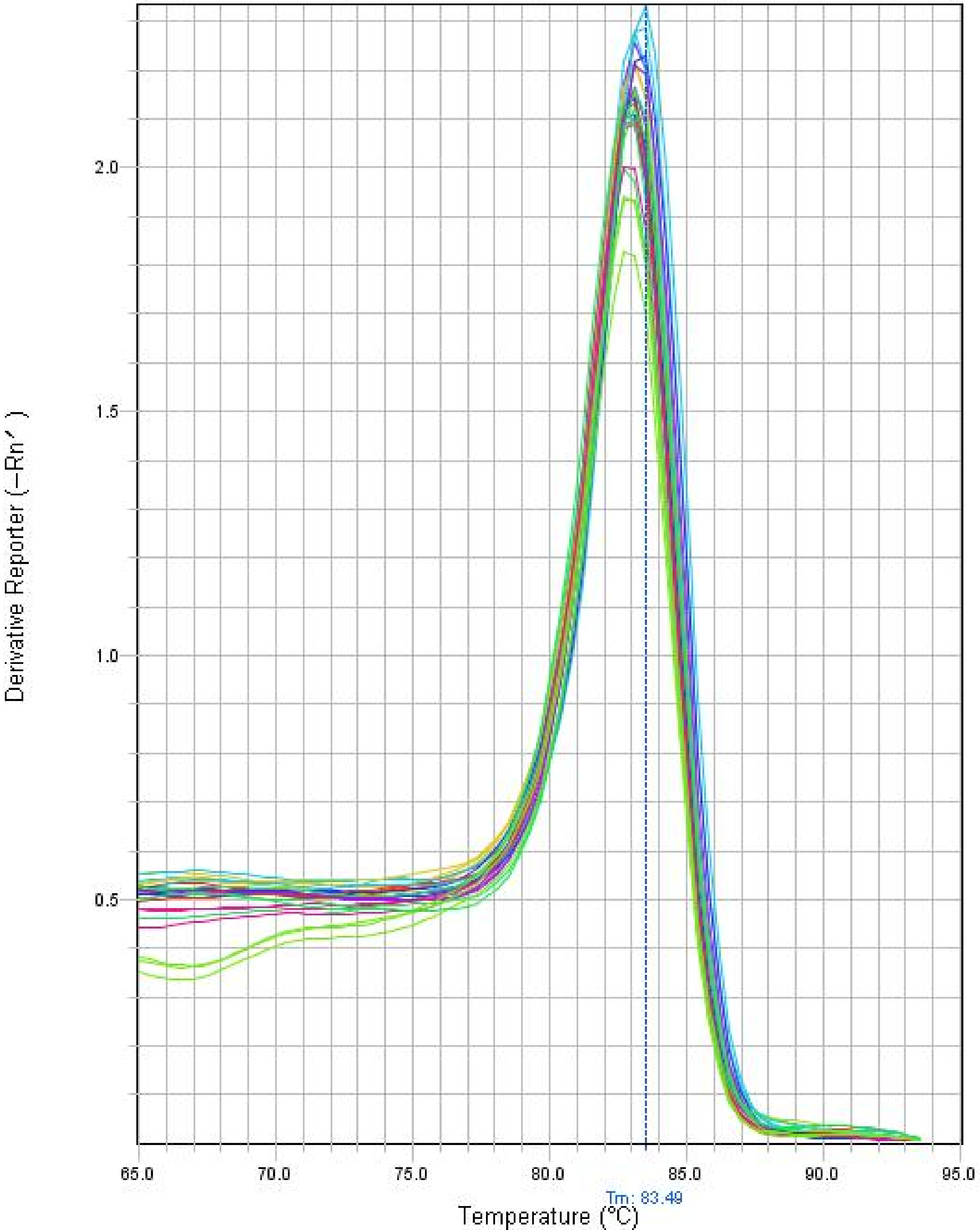
Derivative Reporter (−Rn′)
2.0
1.5
1.0
0.5
65.0
70.0
75.0
80.0
85.0
90.0
95.0
Tm: 83.49
Temperature (°C)
